# Atopy and immune dysregulation among patients with chronic granulomatous disease

**DOI:** 10.3389/fimmu.2025.1739568

**Published:** 2026-01-23

**Authors:** Sophie Eisen, Shani Nagler-Bunker, Matilde Leon-Ponte, Yogi Chopra, Julia Upton, Vy Hong-Diep Kim, Eyal Grunebaum

**Affiliations:** 1 Developmental and Stem Cell Biology Program, The Hospital for Sick Children, Toronto, ON, Canada; 2 The SickKids Food Allergy and Anaphylaxis Program, The Hospital for Sick Children, Toronto, ON, Canada; 3 Division of Hematology/Oncology, The Blood and Marrow Transplant/Cellular Therapy, The Hospital for Sick Children, Toronto, ON, Canada; 4 The Division of Immunology and Allergy, The Hospital for Sick Children, Toronto, ON, Canada

**Keywords:** allergy, atopy, chronic granulomatous disease, hematopoietic stem cell transplantation, inflammatory bowel disease

## Introduction

Chronic granulomatous disease (CGD) is an inborn error of immunity (IEI) that leads to increased vulnerability to recurrent catalase-positive bacterial and fungal infections at an early age (1). CGD is caused by impaired formation of the NADPH oxidase complex, which is critical for effective phagocytosis and removal of intracellular pathogens (2). Defects in the X-linked (XL) *CYBB* gene are considered the most common cause of CGD in North America. Defects in *CYBA*, *NCF1*, *NCF2*, *NCF4* and *CYBC1* genes can cause autosomal recessive (AR) CGD, which may present later in life and with a less severe phenotype (3, 4). CGD diagnosis can be established by demonstrating an abnormal neutrophil oxidative burst and further confirmed by genetic testing (5).

Immune dysregulation including uncontrolled inflammation and granulomas are prevalent in CGD (1). The gastrointestinal tract is most often affected, with up to 50% of CGD patients fulfilling criteria for inflammatory bowel disease (IBD) (6) and many suffering from oral ulcers, anorectal abscesses and fistulas (7, 8). Inflammatory manifestations may impact the genitourinary, pulmonary and ophthalmic systems (9), or lead to hemophagocytic lymphohistiocytosis (HLH) (10). There have also been descriptions of autoimmune abnormalities in patients with CGD, including cutaneous and systemic lupus erythematosus, arthritis, IgA nephropathy, antiphospholipid syndrome, pericardial effusion and type 1 diabetes mellitus (11–13). The pathogenesis of the immune dysregulation in CGD has been attributed to the body’s inefficient clearance of pathogens or the anti-inflammatory roles of NADPH oxidase-derived reactive oxygen species (9, 11, 14, 15). Additional mechanisms proposed for the hyperinflammation in CGD include impaired efferocytosis, reduction in specific toll-like and complement receptors, increased Th17 cells, reduced Nrf2 activity and inflammasome activation (14–17).

Allogeneic hematopoietic stem cell transplants (HSCT) from HLA matched related donors (MRD), matched unrelated donors (MUD), or mismatched related donors (MMRD) can eliminate the susceptibility to infections in CGD (3, 18, 19). However, patients with CGD, particularly with uncontrolled inflammation, are prone to develop graft versus host disease (GVHD) and graft failure (20). Many single and multi-center studies have also shown that allogeneic HSCT can correct and prevent IBD and other inflammatory complications of CGD (3, 6, 7, 18, 19, 21).

Atopic (allergic) manifestations including eczema, allergic rhino-conjunctivitis and asthma, while typically not life-threatening, are associated with significant morbidity. In recent years, the impact of food allergies on patients and families as well as the broader community and health care system has been emphasized. Several primary immune regulatory disorders (PIRD), such as Wiskott-Aldrich syndrome, Immune dysregulation, polyendocrinopathy, enteropathy, X-linked (IPEX) syndrome, CTLA-4 and LRBA deficiencies, defects in the IL-10/IL-10R pathway and others, have been associated with more frequent or difficult to treat inflammation and allergies (22–27). In contrast, CGD is not considered among the classical primary atopic disorders (PAD) where atopy is a prominent feature (28). There have been only a few case reports of atopy, including eczema, among patients with CGD (29, 30). A survey of 33 patients with CGD reported increased frequency of wheezing and itchy rash compared to age-matched siblings (31), while another study of 313 atopic patients with IEI found that 5.9% of them had CGD (32). A recent survey of 30 immunology centers in 23 countries found that 9% of patients with CGD had allergic diseases (33), while a single center study of skin manifestations identified a higher prevalence of eczema in adults with CGD than the population average (34). However, these studies did not characterize the atopic manifestations in CGD along with the immune dysregulation. In addition, the effects of HSCT on atopy in CGD are not known.

Here the hypothesis that CGD is associated with increased atopic and inflammatory manifestations, and that these resolve after HSCT, was tested using a cohort of patients with CGD, which were also compared to patients with other PIRD. The study demonstrates that atopic manifestations, including eczema, are common in CGD and often resolve after HSCT.

## Methods

Data was retrieved from the electronic medical records of patients with CGD and other genetically confirmed PIRD who were diagnosed and managed at The Hospital for Sick Children, Toronto, between January 1^st^, 2000, to July 1^st^, 2024. The study was approved by the institute’s Research Ethics Board (approval #1000064846 and #1000027689). Data was collected from the time of presentation until the last follow-up visit or time of death. For patients who received HSCT, data was also collected at the time preceding HSCT, 2 ± 1 years post-HSCT and 5 ± 1 years after HSCT if available, or until the time of a second HSCT or death. Data collected included the patient’s age at the time of diagnosis, defined as the age when abnormal neutrophil oxidative burst was demonstrated or the age when the IEI was confirmed by genetic sequencing. The current age was defined as the age at the end of the study. Additional characteristics included the CGD gene variant, sex, place of birth (Canada or outside of Canada), living status and whether they received allogeneic HSCT.

The electronic medical records of all patients were reviewed for the presence of physician-diagnosed atopic, inflammatory and autoimmune conditions. The records were also searched for the key terms: ‘eczema’, ‘asthma’, ‘allergy’, ‘allergic rhinitis’, ‘atopic”, ‘atopy’, ‘colitis’, ‘granuloma’ and ‘inflammation’. For each identified atopic or inflammatory condition, the patient’s age at diagnosis, presenting symptoms, imaging and treatment were recorded. Patient-reported conditions and diagnoses that were not medically verified were omitted from analyses. The frequency of atopic and inflammatory manifestations was determined pre-HSCT, including pre-existing conditions at the time of IEI diagnosis and new conditions that developed up until the time of HSCT. For patients with CGD, laboratory features including highest eosinophil counts and IgE concentrations in the blood were noted, together with the upper limit of normal for age.

For patients with CGD, details of HSCT were collected, including donor and stem cell source, conditioning and GVHD prophylaxis regimens. HLA match was defined as complete or nearly complete if no more than one HLA antigen difference was documented. All other donors were defined as mismatched. The HSCT source was also described as from a sibling donor, other related donors such as a parent, or an unrelated donor. Whole blood donor chimerism after HSCT, the percentage of cells that were donor-derived, was recorded at the last available lab measurement. Complete donor chimerism was defined as ≥95% donor-derived nucleated blood cells, while mixed donor chimerism was considered when <95% of cells were donor-derived.

Descriptive statistics were performed to summarize categorical variables as frequencies and continuous variables as means. Comparisons between the CGD and other IEI groups were conducted using Fisher’s exact test for categorical data and unpaired t-test for numerical data using *Prism*. Statistically significant difference was defined as a p-value of less than 0.05.

## Results

### Patient characteristics

The study included 43 patients, 20 with CGD and 23 with other genetically confirmed IEI associated with immune dysregulation. The “other IEI” group included 9 patients with Wiskott-Aldrich syndrome, 6 patients with LRBA deficiency, 3 patients with IPEX syndrome, 2 patients with CTLA-4 deficiency, 2 patients with XIAP/BIRC4 deficiency, and 1 patient with IL-10R deficiency. There were no significant differences in the mean age of IEI diagnosis, sex, birth in Canada, current age, living status and receiving HSCT between patients with CGD and the other IEI group (**Supplementary Table 1**). Most patients with CGD and other IEI studied were below the age of 20 years and had received allogeneic HSCT.

### Atopic and inflammatory conditions among patients with CGD and other IEI

**Figure 1:**
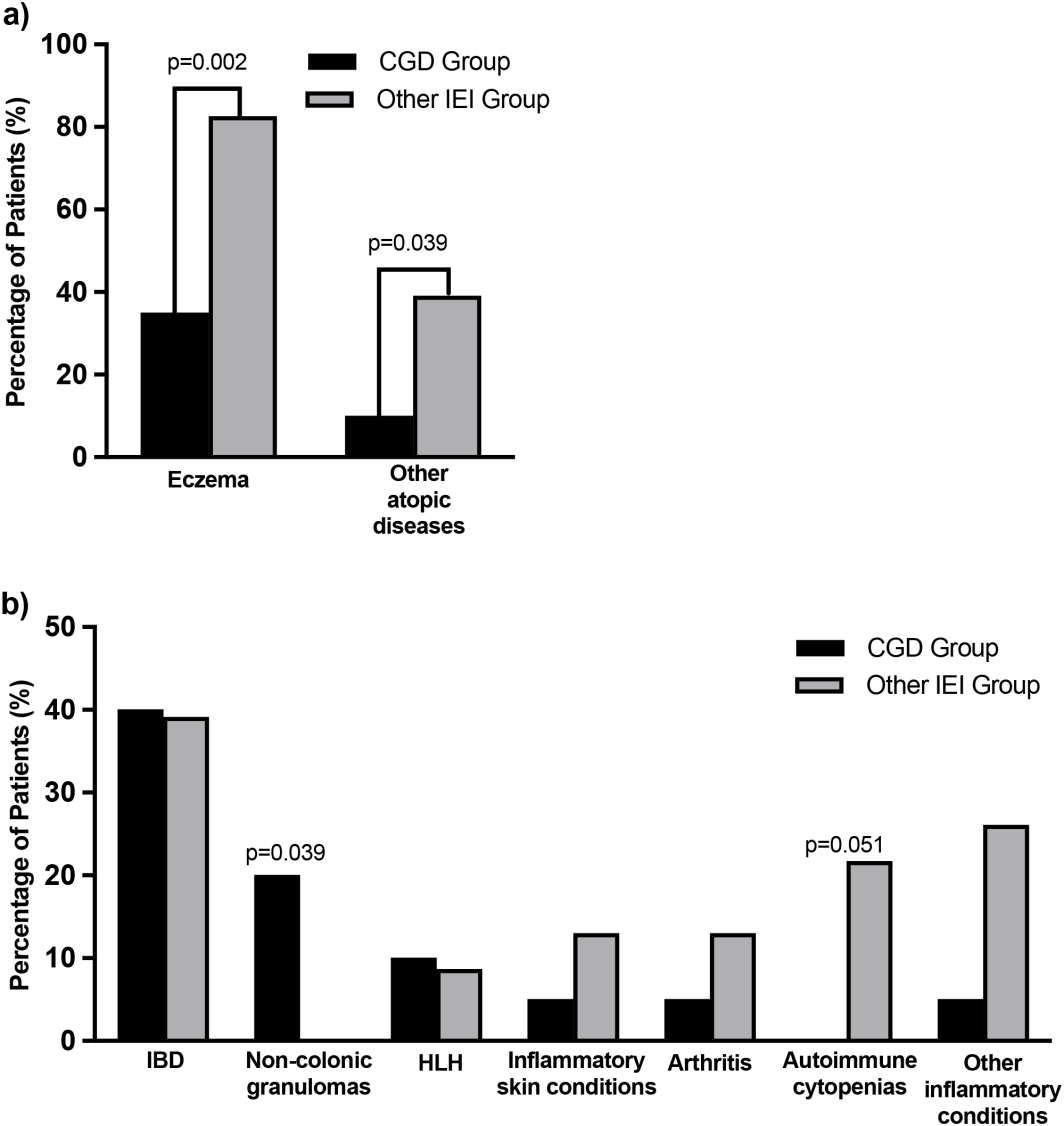
Atopic and inflammatory conditions among patients with CGD and other IEI. The percentage of physician-diagnosed **(a)** atopy and **(b)** inflammatory conditions among patients with CGD and other IEI. Other atopic diseases include allergic rhinitis, asthma and food allergy. IBD = inflammatory bowel disease; HLH = hemophagocytic lymphohistiocytosis. Statistical significance (*p<0.05*) and near statistical significance is noted.

The frequencies of physician-diagnosed atopic, inflammatory and autoimmune conditions were determined for patients with CGD and the other IEI group. Among the 20 patients with CGD, eczema was identified in 7 patients (35%), which was often responsive to topical treatments. Other atopic conditions, such as asthma, allergic rhinitis and food allergies, were identified in only 2 patients (10%). Atopy was frequent among patients with other IEI, as 19 patients (82.6%) and 9 patients (39.1%) of the 23 patients suffered from eczema and other atopic conditions, respectively, which were significantly higher than in the CGD cohort (**Figure 1a**). Inflammatory conditions were reported in 14 of the 23 patients (60.9%) with other IEI and in 9 of 20 patients (45%) with CGD. The inflammatory manifestations, highest eosinophil count and IgE concentration in patients with CGD are detailed in **Supplementary Table 2**. There were no significant differences in the frequency of IBD, HLH, inflammatory skin conditions or other inflammatory diseases between patients with CGD and patients with other IEI (**Figure 1b**). Non-colonic granulomas were significantly more common (p=0.039) among patients with CGD compared to patients with other IEI, while autoimmune cytopenias approached statistical significance (p=0.051). Mild eosinophilia was identified in 6 patients with XL-CGD and 3 with AR-CGD, a difference that was not statistically significant. Only one patient with CGD, of the nine tested, had elevated IgE levels (**Supplementary Table 2**).

### Atopy and inflammatory conditions among patients with CGD caused by different genetic variants

**Figure 2:**
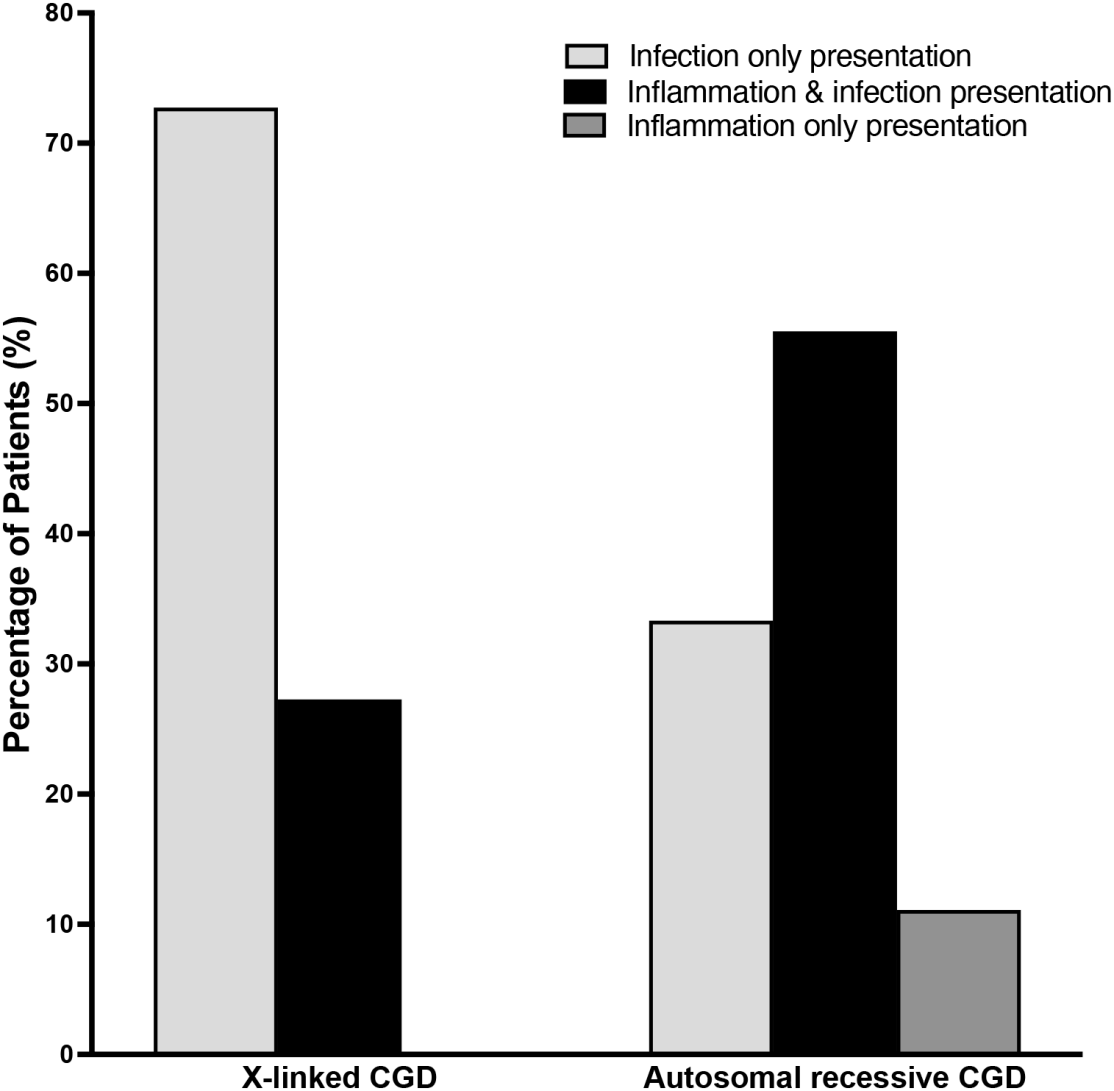
Infection, inflammation or both at the time of diagnosis with CGD. The percentage of patients with X-linked and autosomal recessive CGD that presented with infection only, infection and inflammation, or inflammation only at the time of diagnosis with CGD.

Of the 20 patients with CGD, XL-CGD due to *CYBB* gene variants were identified in 11 patients (55%), while AR-CGD was caused by *NCF1* and *NCF2* variants in 5 and 3 patients, respectively, and a *CYBA* variant in 1 patient. At presentation, inflammation was more frequent in patients with AR-CGD than XL-CGD (**Figure 2**). Six of the 9 patients (66.7%) with AR-CGD presented with inflammation, including 5 patients (55.6%) with concomitant infection while one patient (11.1%) had only inflammation. In contrast, only 3 of the 11 patients (27.2%) with XL-CGD presented with inflammation alongside infection while 8 patients (72.7%) presented exclusively with infection. However, these clinical presentations at CGD diagnosis were not significantly different (p=0.175) between patients with XL- and AR-CGD. During follow-up, a greater proportion of patients with AR-CGD (8 of 9 patients, 88.9%) suffered from inflammatory disease compared to patients with XL-CGD (5 of 11 patients, 45.5%), a difference that approached statistical significance (p=0.073). IBD, HLH and other inflammatory conditions occurred more often among patients with AR-CGD pre-HSCT, but this was not significantly different from patients with XL-CGD (**Table 1**). Similarly, there was no difference in the frequency of eczema and other atopic manifestations between patients with XL-CGD and AR-CGD.

**Table 1:** Inflammation and atopy prior to HSCT among patients with CGD caused by X-linked or autosomal recessive variants.

Condition	X-linked (n=11)	Autosomal recessive (n=9)	p-value*
Inflammation pre-HSCT, n (%)	5 (45.5)	8 (88.9)	0.073
Inflammatory bowel disease, n (%)	3 (27.3)	5 (55.6)	0.362
Hemophagocytic lymphohistiocytosis, n (%)	0 (0)	2 (22.2)	0.285
Non-colonic granulomas, n (%)	2 (18.2)	2 (22.2)	>0.999
Ophthalmic inflammation, n (%)	0 (0)	1 (11.1)	0.450
Arthritis, n (%)	0 (0)	1 (11.1)	0.450
Eczema, n (%)	4 (36.4)	3 (33.3)	>0.999
Other atopic diseases (asthma, allergic rhinitis and food allergy), n (%)	0 (0)	2 (22.2)	0.189

*Comparisons between the X-linked and autosomal recessive CGD groups.

### The effects of hematopoietic stem cell transplantation on atopy, inflammatory and autoimmune conditions among patients with chronic granulomatous disease

**Figure 3:**
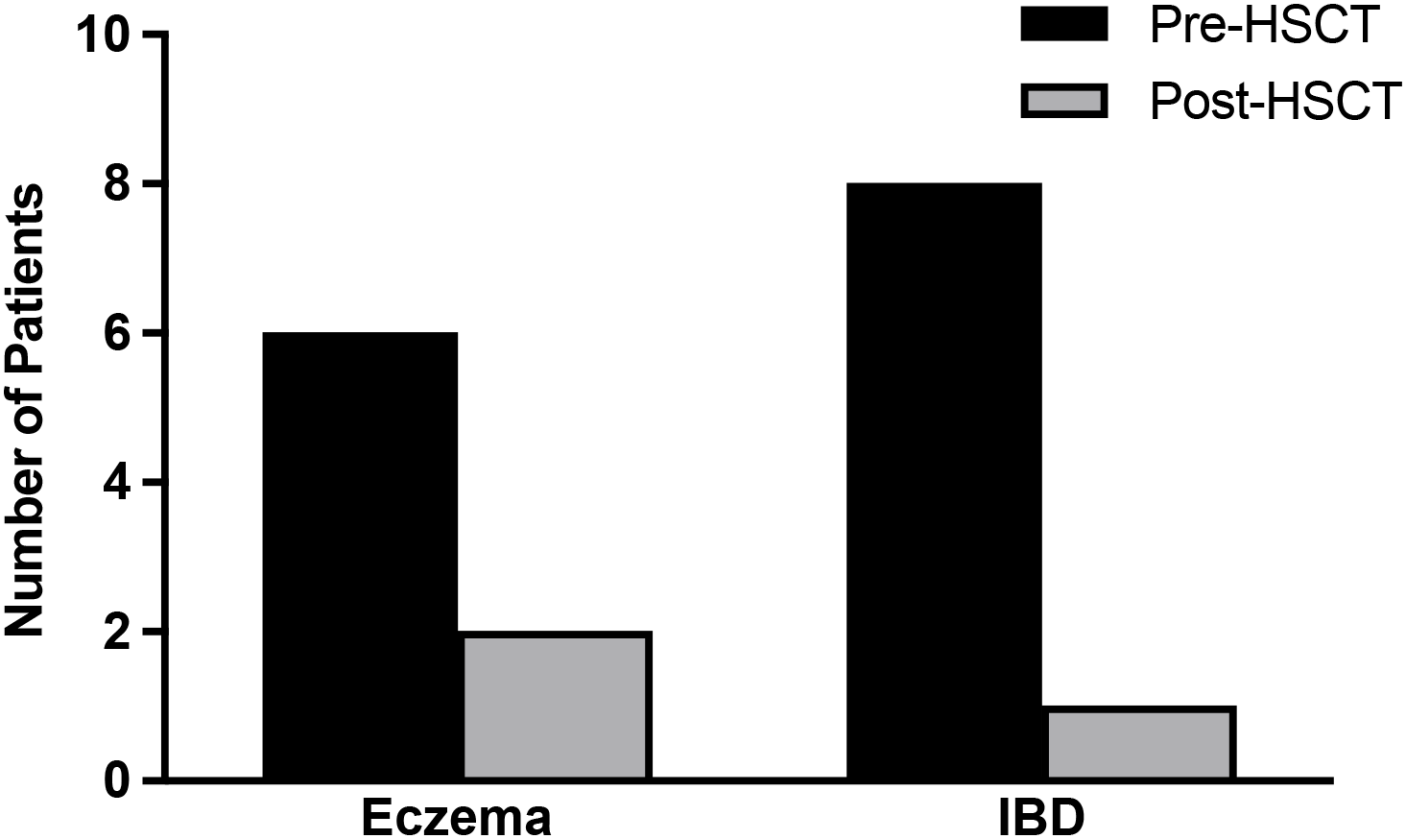
Eczema and inflammatory bowel disease before and after allogeneic hematopoietic stem cell transplantations for CGD. Eczema and inflammatory bowel disease (IBD) pre- and post-allogenic hematopoietic stem cell transplantations (HSCT) in 17 patients with CGD.

Allogeneic HSCT was performed in 17 of the 20 CGD patients (85%) (**Supplementary Table 3**). Of the 17 patients, 6 (35.3%) had eczema before HSCT (**Figure 3**). Eczema resolved in 4 of these patients (66.7%) within 2 ± 1 years after HSCT, reoccurred in one patient after HSCT who had mixed donor chimerism (<95% of cells donor-derived) and could not be evaluated in one patient who had an early graft failure. One patient developed new eczema after HSCT.

Among the 17 CGD patients that underwent allogeneic HSCT, 8 (47.1%) had clinically active IBD before HSCT that was diagnosed by endoscopy or MRI and required treatment with Vedolizumab, 5-ASA and/or corticosteroids. IBD resolved in 7 of the 8 patients (87.5%) at 2 ± 1 years after HSCT. There was no new IBD reported after HSCT. Of the 17 CGD patients that received allogeneic HSCT, 4 patients had granulomas, 2 had HLH, and one suffered from uveitis and optic neuritis before the transplantation. All the autoimmune/inflammatory conditions resolved by 2 ± 1 years after HSCT. New transient autoimmune conditions were noted in 2 patients by 2 ± 1 years post-HSCT, including one who experienced immune thrombocytopenia and another with hemolytic anemia that was treated with corticosteroids. By 5 ± 1 years post-HSCT, one patient developed lichen sclerosis, and another was diagnosed with transient myositis, both requiring corticosteroids treatment. Of these 4 CGD patients with new-onset inflammatory/autoimmune diseases post-HSCT, all had inflammatory conditions before transplantation and 3 suffered from coexistent chronic GVHD.

## Discussion

To investigate the hypothesis that CGD is associated with increased incidence of atopic and inflammatory manifestations, the electronic medical charts of 20 patients with functional and genetically confirmed CGD followed at a single center were interrogated. The results were compared with those of patients with other genetically confirmed IEI, which served as a “control” for our research strategy, and as expected had atopy significantly more common than patients with CGD.

Eczema was found in 35% of our CGD cohort, which is higher than the 15.1% reported among Canadian children (35, 36) and the 10-20% in the general Canadian population (37), yet not unexpected based on the limited data available on eczema in CGD. A previous analysis of atopic conditions found eczema among 18% of 33 patients with CGD, however that study excluded patients younger than 5 years of age and utilized a survey that was completed by patients and parents (31). Similarly, eczema was recorded in 18% of 50 United Kingdom adults with CGD, which was higher than the 2-10% population average (34). In contrast, we included patients across all ages to ensure representation of the different forms of CGD, as XL-CGD tends to present in the first years of life, while AR-CGD often appears later (38).

AR-CGD accounted for 45% of our CGD cohort, which is higher than the 31% reported in a large North American study by the Primary Immunodeficiency Treatment Consortium (PIDTC) (19). This difference might be due to a higher frequency of consanguinity among the diverse population referred to our center. Alternatively, it may be due to increased awareness of referring healthcare providers of the possibility of AR-CGD, including among patients with symptoms that are less typical, such as arthritis, uveitis or HLH. In this study, patients with AR-CGD presented more often with inflammation in comparison to patients with XL-CGD, albeit a difference that was not significantly different. Similarly, the frequency of atopy was not significantly different between XL- and AR-CGD, although studies of larger numbers of patients are required to clarify whether specific genetic CGD variants predispose to atopy.

Furthermore, patients with AR-CGD might have a longer time to develop eczema as patients with XL-CGD are often ushered sooner to definitive treatment (19). Additionally, we relied on objective diagnosis and documentation of atopy by health care providers, thereby circumventing potential recall biases, and collected the information directly from patients’ charts rather than subjective data entry by providers. The latter might explain why 5% or less of the 515 patients with CGD registered in the USIDNET database were reported to have atopic dermatitis/eczema, asthma, or allergic rhinitis, and none with food allergies (39).

In contrast to eczema, other atopic conditions were not common among patients with CGD and occurred less frequently compared to the patients with other IEI associated with inflammation. The difference is likely not due to reporting bias, as the frequencies of inflammatory manifestations identified in the patients’ charts were similar in both groups. Several factors might have contributed to the higher frequency of eczema in CGD, but not other atopic manifestations. Patients with CGD often suffer from nutritional deficiencies and receive antimicrobial and immune suppressive medications to control infections and inflammation, respectively. This might cause skin microbial dysbiosis, as it does for the gut microbiome (40). Indeed, the skin of patients with atopic dermatitis is characterized by reduced microbial diversity and overrepresentation of *Staphylococcus aureus* (41), as also commonly found in patients with CGD (42). Neutrophils from CGD patients were shown to have decreased expression of the CD11b/CD18 complement receptor in one study, which is associated with impaired phagocytosis of *S. aureus* (43). This may contribute to *S. aureus* skin colonization, which could perpetuate the skin microbial dysbiosis found in atopic dermatitis (44). Alternatively, impaired ability to clear apoptotic cells (efferocytosis) (4), or the abnormal production of oxidative metabolites in CGD, which are important in quenching ongoing inflammation, might contribute to the persistent process as previously demonstrated in the skin of patients with CGD (45). It is also possible that the differences in atopy between patients with CGD and the other PIRD are due to differences in the mechanism of diseases. Development of atopy in patients with IPEX, Wiskott Aldrich Syndrome and the other IEI is mediated by abnormal T and B regulatory cells (46, 47), while in CGD there are predominantly Th1 and Th17 responses (48, 49). Heightened Th17 responses in CGD may occur by several means, such as impaired signaling through the redox-sensitive anti-inflammatory regulator Nrf2 and increased NLRP3 inflammasome activation (16). Notably, in the NADPH oxidase (Nox)2-deficient mice there is exaggerated asthma driven by enhanced Th2 differentiation and effector function (50). Regardless of the mechanism, the data presented here suggest that patients with CGD are prone to eczema, and that frequent examination of their skin is warranted, particularly as the disrupted skin barrier can further increase the risk of local and systemic infections (51, 52).

In our cohort, 65% and 40% of patients with CGD suffered from inflammatory conditions and IBD, respectively, which is higher than the 40% and 30% reported recently by the PIDTC (19). These differences are possibly due to the increased proportion of patients with AR-CGD in our cohort, as this group suffered from inflammatory features more commonly than patients with XL-CGD. The differences in inflammatory manifestations might indicate that while XL and AR-CGD are often grouped together due to the common disruption of NADPH, there are additional mechanistic differences resulting from the specific gene product affected. Non-colonic granulomas, found in 4 patients with CGD, were absent among patients with other IEI, which is not surprising as granulomatous formation is one of the hallmarks of CGD (53). HLH was recorded in 2 of the CGD patients, a rare inflammatory condition that has been reported previously in patients with CGD (10, 54–57). Similarly, arthritis and uveitis have been uncommonly reported in CGD (11, 58). The data presented here emphasizes the diverse inflammatory manifestations that can occur in CGD, and the need to maintain a high index of clinical suspicion in patients with early-onset inflammatory diseases with or without unusual infections.

Most (85%) of our patients with CGD received allogeneic HSCT, a proportion that is higher than that in many multi-center large cohort reports (6, 59, 60). This difference might reflect variability between health care providers in utilizing definitive treatment for CGD, as some centers have been reluctant in offering HSCT for CGD until recent years. Studies of large groups of patients with CGD have shown that allogeneic HSCT can effectively alleviate infections and inflammatory conditions such as IBD (3, 19, 21, 61). In addition, we demonstrate for the first time that HSCT can also lead to the long-term resolution of eczema, further emphasizing the role of immune dysregulation in this atopic manifestation. The small number of patients with CGD with eczema after HSCT limits our ability to assess the potential contribution of mixed donor chimerism to atopy, which will require studying larger cohorts.

While our study emphasizes that CGD itself *can* present with atopy and immune dysregulation and drawing attention to the high prevalence of eczema in CGD, it has several limitations due to its retrospective design. For example, the severity of the eczema was not routinely evaluated by scoring systems commonly used in clinical practice, such as the Scoring for Atopic Dermatitis Index or the Eczema Area and Severity Index. Similarly, the diagnosis of asthma and food allergies for most patients did not include pulmonary function testing, or specific IgE to food and skin prick testing. Details of eczema among family members were also not systematically recorded, which may have illuminated polygenic atopy risk.

Nevertheless, the study presented here extends the range of immune dysregulation in CGD to include eczema, possibly through mechanisms that differ from the typical inflammatory conditions seen in CGD. Clinicians should perform routine skin examinations and consider pulmonary function tests and allergy testing in patients suffering from CGD with relevant symptoms. Moreover, the impaired skin barrier in patients with CGD and eczema may increase the susceptibility to secondary infections, hence clinicians may need to incorporate early dermatologic management into standard CGD care. The study also highlights the benefits of allogeneic HSCT in alleviating eczema in CGD, further supporting the use of definitive treatments for the correction of diverse immune dysregulation in CGD.

## Data Availability

The original contributions presented in the study are included in the article/**Supplementary Material**, further inquiries can be directed to the corresponding author/s.

## Supplementary material

Supplementary Table 1 Characteristics of the patients with chronic granulomatous disease (CGD) and other inborn errors of immunity (IEI).

Supplementary Table 2 Presenting infections and inflammation/autoimmunity at the time of CGD diagnosis.

Supplementary Table 3 Characteristics of the allogeneic hematopoietic stem cell transplantation in 17 patients with CGD.
