# Supplementary material for: Atopy and immune dysregulation among patients with chronic granulomatous disease
Source: Front Immunol. 2026 Jan 23;16:1739568. doi: 10.3389/fimmu.2025.1739568 (PMC12876255; doi:10.3389/fimmu.2025.1739568)
Supplement: Supplementary Table 1 — Characteristics of the patients with chronic granulomatous disease (CGD) and other inborn errors of immunity (IEI). [file Table1.docx]

**Supplementary data**

**Supplementary Table 1:** Characteristics of the patients with chronic granulomatous disease (CGD) and other inborn errors of immunity (IEI)

| Characteristic | CGD Group (n=20) | Other IEI Group (n=23) | p-value* |
| --- | --- | --- | --- |
| Mean age of IEI diagnosis (years) | 3.892 | 5.967 | 0.235 |
| Male n (%) | 14 (70) | 16 (69.6) | >0.999 |
| Birth in Canada | 16 (80) | 17 (73.9) | >0.999 |
| Current age between 1 to 10 years, n (%) | 10 (50) | 8 (34.8) | 0.597 |
| Current age between 11 to 20 years, n (%) | 6 (30) | 12 (52.2) |  |
| Current age >20 years, n (%) | 4 (30) | 3 (13.0) |  |
| Currently alive | 19 (95) | 22 (95.7) | >0.999 |
| Allogeneic hematopoietic stem cell transplantation | 17 (85) | 14 (60.9) | 0.099 |

*= The CGD and other IEI groups were compared using Fisher’s exact test for categorical data and unpaired t-test for numerical data.

**Supplementary Table 2:** Presenting infections and inflammation/autoimmunity at the time of CGD diagnosis.

| Patient No. | Inheri-tance | Presenting infection | Organism | Presenting inflammation/ autoimmunity | IgE* level* (IU/mL) | Eosinophils* (x 10^9^/L) |
| --- | --- | --- | --- | --- | --- | --- |
| 1 | XL | Necrotizing pneumonia; UTI | Candida | None identified | <25 (440) | **1.39 (0.97)** |
| 2 | XL | Bacteremia; Nasal swab positive | Moraxella nonliquefaciens; Boca virus, Rhinovirus | None identified | ND | 0.12 (0.50) |
| 3 | XL | Liver abscesses | None identified | IBD | ND | **1.12 (0.50)** |
| 4 | XL | Liver abscesses | Staphylococcus aureus | Liver granuloma | ND | **1.26 (0.7)** |
| 5 | XL | Fever of unknown origin | Norovirus | IBD; Possible CGD-related vasculitis | ND | 0.45 (0.79) |
| 6 | XL | Perianal abscess and fistula | None identified | None identified | <25 (440) | **1.19 (0.5)** |
| 7 | AR | Pneumonia | Strepto. fulminans, Acinetobacter, Enterovirus, Bocavirus | None identified | ND | **0.59 (0.53)** |
| 8 | XL | Neck abscess | None identified | None identified | <25 (440) | 0.3 (0.44) |
| 9 | AR | Pneumonia | Aspergillosis fumigatus | Migratory arthritis Liver granuloma | 208 (440) | **0.91 (0.38)** |
| 10 | XL | Mediastinal and hilar lymphadenitis | Aspergillus | None identified | **2151 (90)** | **0.72 (0.5)** |
| 11 | AR | Liver abscess | Unknown | None identified | ND | 0.06 (0.5) |
| 12 | XL | Cervical lymphadenitis | None identified | None identified | 74 (440) | 0.62 (0.7) |
| 13 | AR | Splenic abscess | None identified | HLH | ND | 0.39 (0.7) |
| 14 | AR | Bacteremia; Splenic abscess | Burkholderia cepacia | HLH | <25 (440) | 0.23 (0.7) |
| 15 | AR | Cervical lymphadenitis | Staphylococcus aureus | IBD; Cervical lymph node necrotizing granuloma | ND | 0.74 (0.97) |
| 16 | AR | Pneumonia | Burkholderia cepacia, Bocavirus | None identified | ND | 0.42 (0.97) |
| 17 | AR | Necrotizing enterocolitis | None identified | SIRS | 39 (440) | **1.31 (0.79)** |
| 18 | AR | None identified | None identified | Uveitis; Splenic granulomas | 54 (440) | 0.19 (0.97) |
| 19 | XL | Sepsis; Auricular lymphadenitis | Aspergillus | None identified | 9 (12) | **1.38 (0.5)** |
| 20 | XL | Pulmonary infiltrate, cervical lymphadenitis | None identified | None identified | ND | 0.11 (0.5) |

XL = X-linked; AR = autosomal recessive; HLH = hemophagocytic lymphohistiocytosis; UTI = urinary tract infection; IBD = inflammatory bowel disease; SIRS = systemic inflammatory response syndrome. ND=not done.

*- Highest value prior to HSCT (upper limit of normal for age). Bolded are abnormal results.

**Supplementary Table 3:** Characteristics of the allogeneic hematopoietic stem cell transplantation in 17 patients with CGD.

| Mean age at time of HSCT (years) | 5.279 |
| --- | --- |
| Donor type^a^ and HLA match, n (%) |  |
| MUD | 8 (47.1) |
| MSD | 4 (23.5) |
| MMUD | 3 (17.6) |
| MMRD | 2 (11.8) |
| Stem cell source, n (%) |  |
| Bone marrow | 8 (47.1) |
| Peripheral blood stem cells | 6 (35.3) |
| Umbilical cord blood | 3 (17.6) |
| Conditioning regimen, n (%) |  |
| Treo-Flu-Cy-ATG | 12 (70.6) |
| Busulfan and Cyclophosphamide | 5 (29.4) |
| GVHD prophylaxis, n (%) |  |
| Cyclosporin A and Steroids | 5 (29.4) |
| Tacrolimus and Methotrexate ± Other | 6 (35.3) |
| Tacrolimus and Mycophenolate mofetil | 2 (11.8) |
| Tacrolimus and Mycophenolate mofetil and post-transplant cyclophosphamide | 1 (5.9) |
| Mycophenolate mofetil and post-transplant cyclophosphamide | 1 (5.9) |
| Tacrolimus and Steroids | 2 (11.8) |
| Donor chimerism at last available measurement, n (%) |  |
| Full | 11 (64.7) |
| Mixed | 6 (35.3) |

^a^MUD = matched unrelated donor; MSD = matched sibling donor; MMUD = mismatched unrelated donor; MMRD = mismatched related donor; Treo = treosulfan; Flu = fludarabine; Cy = cyclophosphamide; ATG = antithymocyte Globulin; GVHD = graft versus host disease.
